# The Physical, Psychological, and Social Day-to-Day Experience of Women Living With Endometriosis Compared to Healthy Age-Matched Controls—A Mixed-Methods Study

**DOI:** 10.3389/fgwh.2021.767114

**Published:** 2021-12-15

**Authors:** Elisabeth Olliges, Alina Bobinger, Annemarie Weber, Verena Hoffmann, Timo Schmitz, Roxana M. Popovici, Karin Meissner

**Affiliations:** ^1^Faculty of Medicine, Institute of Medical Psychology, LMU Munich, Munich, Germany; ^2^Division of Health Promotion, Coburg University of Applied Sciences, Coburg, Germany; ^3^Department of Psychosomatic Medicine and Psychotherapy, Barmelweid, Switzerland; ^4^Chair of Epidemiology, University of Augsburg, Augsburg, Germany; ^5^Kïz, Munich, Germany; ^6^Department of Gynecologic Endocrinology and Fertility Disorders, Heidelberg University Women's Hospital, Heidelberg, Germany

**Keywords:** endometriosis, mixed-methods design, everyday-experiences, symptom diary, interviews, bio-psycho-social model

## Abstract

**Background:** Endometriosis is characterized by lesions of endometrial tissue outside the uterus. Chronic pain is considered as main symptom, but challenges can relate to various physical, mental, and social aspects of the women's lives. The aim of our study was to gain a holistic understanding of the everyday reality of women with endometriosis compared to healthy controls.

**Methods:** The total sample comprised 12 hormone-free endometriosis patients (EP) and 11 age-matched healthy women (HC). A mixed-methods design was used comprising semi-structured interviews, standardized questionnaires and a comprehensive diary to assess pain ratings and various mental and physical symptoms over the course of a menstrual cycle. Interviews were recorded, transcribed, and evaluated according to phenomenological analysis using the MAXQDA software.

**Results:** Interviews showed that living with endometriosis was associated with an impairment in everyday life. Physical strains, especially pain, high levels of psychological distress, and social limitations have been reported. Living with endometriosis affected the patients' personality and they “no longer felt like themselves.” Physical and psychological symptoms were reported to interfere with social interaction and participation. Evaluation of the standardized questionnaires revealed significant impairments in EP compared to HC in regard to anxiety and depression scores (both *p* < 0.001; Hospital Anxiety and Depression Scale), mental and physical quality of life (both *p* < 0.001; Short-Form Health Survey-12), stress ratings (*p* < 0.001; Patient Health Questionnaire-15) and functional well-being (*p* < 0.001; Functional Well-being-7). The highest levels of mean pelvic pain and dyschezia were observed in EP during menstruation, but mean pain ratings and dyschezia were increased in EPs compared to HP during the whole cycle. EP reported mental symptoms (e.g., depressed mood or anxiety) mainly during menstruation, while HC did not show any mental symptoms during the cycle. In addition, physical symptoms were elevated during the entire cycle in EPs (all *p* < 0.01).

**Discussion:** The mixed-methods approach enabled to interpret the interviews, the standardized questionnaires, and the symptom diary in a broader context of everyday life. The symptoms do not appear to act independently, but rather influence each other. This leads to a complex interplay of physical, mental, and social impairments, with pain often being the starting point.

## Introduction

Endometriosis is a complex, inflammatory disease, characterized by lesions of endometrial tissue outside the uterus (1–4). Endometriosis has an estimated prevalence of 6–15% of women in their reproductive age worldwide (5–7). The etiology of endometriosis is not fully understood (8). There are several hypotheses regarding the etiopathogenetic mechanisms of endometriosis; the most widely accepted transportation theory assumes that viable endometrial cells circulate back during menstruation through the tubes (menstrual reflux) and cluster at neighboring pelvic structures (9). In addition to a presumed multifactorial etiology, which is not yet fully understood, and the lack of a curative treatment, there are many other unanswered questions (10, 11). Further challenges include the diagnostic process, which requires laparoscopic procedures, and the often massively delayed diagnosis (8 years on aver-age) (12, 13).

Endometriosis manifests at various levels, e.g., anatomic, histologic, or immunologic (3), as well as with a variety of different symptoms (e.g., chronic fatigue or bladder- and bowel-associated symptoms) and comorbidities (e.g., depression) (14–17). Chronic pain (e.g., pelvic pain, dysmenorrhea, or dyspareunia) and infertility are considered the main symptoms (17), but the complaints faced by women with endometriosis may relate to various physical, emotional, and social aspects of the women's daily lives (10, 14, 18–20). Current treatment approaches for pain and infertility (e.g., hormonal drugs or repeated surgical procedures) are not always effective and are often associated with unpleasant side effects (21, 22). In addition, there is no direct association between histologic findings and disease symptoms, and disease progression is difficult to predict (3, 23).

Several studies have reported reduced quality of life in women with endometriosis, characterized by constraining physical conditions as well as negative emotions such as uncertainty, anxiety, or depressive symptoms (14, 16, 18, 24). In most of these studies, patient-reported outcomes were measured at only a single time point during the menstrual cycle and focused on either mental or physical well-being, mostly related to pain (25–28). Cross-sectional studies bear the risk of recall bias or loss of relevant information. There is insightful research that examines pain using an electronic diary to gather information about the “day-to-day” experience (23–25), which seems to be a worthwhile method to show the course of symptoms over an entire menstrual cycle.

The aim of the present study was to gain a holistic understanding of the everyday condition of (hormone-free) women with endometriosis. In order to meet the complexity of the research question, method triangulation was used, i.e., both quantitative and qualitative approaches were applied (29). To our knowledge, this is the first mixed-methods study to collect data from women with endometriosis on a daily basis over the course of a menstrual cycle.

Comprehensive pain ratings (e.g., pelvic pain, dyspareunia, dyschezia) were assessed. In addition, since endometriosis patients have been shown to suffer not only from pain, but also from numerous physical symptoms as well as mental problems such as depression or anxiety, we also assessed the symptoms of premenstrual dysphoric disorder (PMDD) on a daily basis (30). PMDD symptoms comprise depressed mood, anxiety, anger, emotional instability, loss of interest, lethargy, difficulty concentrating, change in appetite, hypersomnia/insomnia, perceived loss of control, and physical symptoms (breast discomfort, joint or muscle pain, a sensation of “bloating” or weight gain) (31). Diary data were supplemented with data from standardized questionnaires collected during the menstrual phase: The Hospital Anxiety and Depression Scale (HADS) (32), State and Trait Anxiety Inventory (STAI) (33, 34), 12-Item Short-Form Health Survey (SF-12) (35), the somatic and stress scales from the Patient Health Questionnaire (PHQ-15) (36), and Functional Well-being (FW-7) (37). In order to better understand the interplay of the individual symptoms and to learn more about the individual impact of the physical and mental symptoms on the women's everyday life, additional semi-structured interviews were conducted with the endometriosis patients.

## Methods and Materials

### Study Design and Sample

A prospective observational study was performed in 12 endometriosis patients (EP) and 11 age-matched healthy controls (HC). A mixed-methods design was applied, comprising semi-structured interviews, standardized questionnaires, and a comprehensive diary to assess pain ratings and PMDD symptoms (30). The study also included the collection of menstrual and venous blood to evaluate immunological changes [results reported elsewhere (38)]. The study protocol was approved by the ethical committee of the Medical Faculty at LMU Munich (no. 17-695) and the research was performed in accordance with the principles of GCP/Declaration of Helsinki. All study participants gave written informed consent.

Between January 2018 and August 2019, women with histologically confirmed endometriosis and age matched healthy volunteers without endometriosis or menstrual pain were recruited. Inclusion criteria were the following: Age between 18 and 45 years, regular menstrual cycle and sufficient knowledge of German language. Further inclusion criteria for the patients with endometriosis was biopsy-confirmed endometriosis. An additional criterion was related to maximum pelvic pain during the last three menstrual cycles (dysmenorrhea) assessed by a numeric rating scale (0 = no pain, 10 = worst pain imaginable): Inclusion criterion was 5 or more for patients with endometriosis, and 3 or less for healthy controls. All participants underwent gynecological examination in order to confirm (EP) or exclude (HC) the presence of endometriosis. For endometriosis patients, the stage of the disease according to the Revised Classification of the American Society for Reproductive Medicine (rASRM) (39) was assessed by reviewing the patients' medical chart and confirmed/adapted during the gynecological examination. Exclusion criteria for both groups were the use of psychotropic drugs and/or manifest mental illness, a malignant disease, the acute need of treatment of a gynecological disease, medication with hormonal drugs, pregnancy, and current breastfeeding.

### Procedure

EP were recruited through outpatient centers, and HC by postings at local universities. In a detailed telephone call, the aim and procedure of the study, voluntary participation, and data protection were explained. Furthermore, the inclusion and exclusion criteria were ascertained and any open questions were answered. After signing informed consent, all participants completed an online questionnaire that collected information regarding demographics and disease history, resp. health status. Already at the beginning of the study, it was clearly communicated to the participants when the expected onset of the menstruation and thus the start of the diary, as well as the start of the second menstruation and the visit to the institute for the interviews would take place. Participants informed the research team at the first day of their next menses and received an email that included a link to a comprehensive survey to assess pain levels and PMDD symptoms. On each day during the menstrual cycle, participants received a similar email at the same time of day, depending on the time of their first diary response. If a participant did not respond within 12 h, a reminder email was automatically sent via the RedCAP distribution tool (40, 41). RedCAP was set up in such a way that every question had to be answered before the questionnaire could be sent off, so that there was hardly any missing data. At the time of their second menstruation, participants were invited to the Institute of Medical Psychology (LMU Munich) to conduct the semi-structured interviews face-to-face, and to complete a catalog of standardized questionnaires. Healthy control subjects received an expense compensation of 50 €.

### Material and Measures

#### Questionnaires

Standardized and widely used questionnaires were completed during the menstrual phase to assess depression and anxiety (HADS, STAI) (32–34), mental and physical quality of life (SF-12) (35), physical symptoms and stress (PHQ-15) (36), and well-being (FW-7) (37).

#### Symptom Diary

On a daily basis, participants rated their mean and maximum pelvic pain, maximum alguria, maximum dyschezia, and maximum dyspareunia on numeric rating scales (NRS, 0 = no pain, 100 = maximum pain). In addition, the German Premenstrual Symptom Inventory was applied to assess 27 symptoms that are frequently observed during a menstrual cycle (42). Symptoms were assessed on 6-point Likert scales (0 = not at all; 5 = extreme) (43) and mean scores were computed for the following domains: depressed mood, anxiety, tension, emotional instability, loss of interest, lethargy, fatigability, change in appetite, hypersomnia/insomnia, loss of control, and physical symptoms (breast discomfort, joint or muscle pain, a sensation of “bloating” or weight gain). There were further questions about the need of drugs (name and dosage of the drug), the presence of menses or metrorrhagia (yes/no), and bleeding intensity (NRS, 0 = no bleeding; 100 = maximum bleeding).

#### Interviews

The development of the semi-structured interview guide was based on a literature review and allowed to conduct the interviews as standardized as possible, while at the same time leaving enough space for individual answers. Interviews were conducted face-to-face and were audio-recorded, transcribed and evaluated using the MAXQDA software (44, 45). To preserve the integrity of the women, confidential data such as names or location information were anonymized during transcription and pseudonyms with consecutive identification numbers were used. Data analysis was performed by two researchers (AW and AB) using an interpretive-hermeneutic phenomenology approach. The interviews lasted an average of 23.17 min.

### Statistical Analyses

Quantitative data was analyzed by means of IBM SPSS Statistics 25, with a *p* ≤ 0.05 considered as significant. To compare baseline characteristics as well as questionnaire scores between EP and HC, independent-samples *t*-tests, Pearson's chi-square-tests and Mann-Whitney-*U*-Tests were performed, as appropriate. Diary data were analyzed by non-parametric statistics. As the length of the women's cycles varied, the diary data was grouped into three phases in order to enable better comparability: The menstruation phase (MP; the individual mean value of all days during menstruation), the premenstrual phase (PMP; mean value of the 5 days prior to menstruation) and the intermediate phase (IP; mean value of the days between the menstruation phase and the pre-menstruation phase) (31, 46, 47). Pain ratings and PMDD symptoms were compared between EP and HC for each cycle phase using Mann-Whitney *U*-tests (*p*-values were Bonferroni-corrected for the three cycle phases). Within-subject changes in pain ratings and PMDD symptoms between cycle phases were explored using Wilcoxon signed-rank tests (with uncorrected *p*-values).

## Results

Thirteen patients and 15 healthy subjects were eligible for the study. One patient had to be excluded because of a planned medical infertility treatment. Two healthy controls had to be excluded because they were too young to be matched with a patient. Two healthy controls reported significant menstrual pain during the observation period and were excluded, as well. Finally, 11 patients and 12 healthy controls were included in the study.

EP and HC were comparable in regard to age, body mass index, relationship status, and educational background (Table 1). In EP, mean time since the first diagnostic surgery was 41.92 months (±17.74 SD) and the rASRM score ranged between I and IV (Table 1).

**Table 1:** Socio-demographic, health-related, and diagnostic characteristics of study participants.

**Variables**	**EP (*n =* 12)**	**HC (*n =* 11)**	***p-value***
Age (years), *mean (SD)*	33.67 (6.31)	29.14 (6.77)	0.16^a^
BMI, *mean (SD)*	21.63 (2.03)	22.04 (4.26)	0.48^a^
**Educational background**, ***n (%)***			
Secondary school	1 (8.25)	0	
High school	4 (33.33)	0	
A-level	4 (33.33)	3 (27.27)	
University degree	3 (25)	8 (72.72)	0.06^b^
**Relationship status**, ***n*** **(%)**			
Single	3 (25)	1 (9.1)	
In a relationship (not married)	7 (58.33)	8 (72.72)	
Married	2 (16.67)	2 (18.18)	0.6^b^
Months since first endometriosis diagnosis, *mean (SD)*	41.92 (17.74)		
**Endometriosis stage, ASRM Score**, ***n*** **(%)**			
I	3 (25)	–	
II	3 (25)	–	
III	3 (25)	–	
IV	3 (25)	–	
**Depression, Anxiety, Stress (during menstruation)**, ***mean (SD)***			
Depression (HADS)	6.25 (3.67)	3 (3.13)	0.03^c^
Anxiety (HADS)	7.75 (3.1)	4.45 (2.42)	0.01^c^
State anxiety (STAI)	42.82 (4.26)	39.78 (5.29)	0.17^c^
Stress scale (PHQ)	7.25 (3.91)	3 (1.84)	<0.001^c^
**Physical symptoms and Quality of Life (during menstruation)**, ***mean (SD)***			
Physical symptoms scale (PHQ)	8.25 (3.22)	1.64 (1.21)	<0.001^c^
Physical health sum score (SF-12)	43.96 (9.06)	55.92 (2.25)	<0.001^c^
Mental health sum score (SF-12)	39.01 (10.74)	51.09 (4.15)	<0.001^c^
Habitual Well-being (FW-7)	22.08 (6.37)	27.91 (4.78)	0.02^c^

*EP, endometriosis patients; HC, healthy controls; SD, standard deviation; BMI, body mass index; HADS, Hospital Anxiety and Depression Scale; STAI, State and Trait Anxiety Inventory; PHQ, Patient Health Questionnaire; IQR, interquartile range; SF-12, 12-Item Short-Form Health Survey; FW-7, Marburger Fragebogen zum habituellen Wohlbefinden; ASRM, American Society for Reproductive Medicine*.

a *Two-sided independent t-test*,

b *Pearson's Chi-Square-Test*,

c *Mann-Whitney-U-Test*.

### Standardized Questionnaires

Non-parametric tests evaluating the results of the *standardized questionnaires* revealed significant differences between EP and HC in regard to anxiety and depression scores (both *p* < 0.05; HADS), mental and physical quality of life (both *p* < 0.001; SF-12) as well as physical symptoms and stress ratings (both *p* < 0.001; PHQ) and functional well-being (*p*< *0.0*5; FW-7), with EPs showing higher burden and worse quality of life and well-being than HC. No significant group differences were found for state anxiety (STAI; *p* = 0.17) (Table 1).

### Symptom Diary

As shown in Table 2, the highest levels of mean and maximum pelvic pain and dyschezia were observed in EP during the menstruation phase. Maximum pain ratings were significantly higher in the intermediate phase than in the premenstruation phase only in EPs. HC reported mild pelvic pain only during the menstruation phase. Group comparisons indicated higher ratings of mean and maximum pain and dyschezia in EP compared with HC during all three phases of the menstrual cycle. In addition, maximum alguria was elevated in HC during the intermediate phase compared with the menstruation phase but still lower than in EP (Table 2).

**Table 2:** Diary results for pain ratings in endometriosis patients and healthy controls, according to cycle phases.

**Pain ratings, NRS 0-100 (0 = no pain; 100 = maximum pain)**	**Group (EP *n =* 12; HC *n =* 11)**	**Phase**		
		**Menstruation phase**	**Intermediate phase**	**Premenstruation phase**
Mean pelvic pain. *median (IQR)*	EP	42.34 (17.63; 52.95)^***^	10.32 (3.51; 23.88)^***^	12.2 (1.8; 21.8)^*^
		≤ 0.01*^a^*	≤ 0.001*^a^*	n.s.
	HC	5 (0.5; 8.4)	0 (0; 0.2)	0 (0; 1.17)
		≤ 0.05*^a^*	≤ 0.05*^a^*	n.s.
Maximum pelvic pain. *median (IQR)*	EP	80.00 (70; 94)^***^	52 (40; 70.75)^***^	27.5 (3.75; 42.5)^*^
		≤ 0.001*^a^*	≤ 0.01*^a^*	≤ 0.001*^a^*
	HC	23 (2; 50)	3 (1; 10)	0 (0; 2)
		≤ 0.05*^a^*	≤ 0.05*^a^*	n.s.
Maximum dyschezia. *median (IQR)*	EP	17.85 (1.8; 23.05)^***^	5.19 (2.44; 22.52)^**^	5 (0.45; 26.25)^**^
		n.s.	n.s.	n.s.
	HC	0 (0; 0)	0 (0; 0)	0 (0; 0)
		n.s.	n.s.	n.s.
Maximum alguria. *median (IQR)*	EP	1.4 (0; 8.2)	2.95 (0.92; 6.8)^**^	1 (0; 3)
		n.s.	n.s.	n.s.
	HC	0 (0; 0.83)	0.45 (0; 1.6)	0 (0; 0)
		n.s.	n.s.	≤ 0.05*^a^*
Maximum dyspareunia. *median (IQR)*	EP	0 (0; 0)	3.75 (0; 9.5)	0 (0; 0)
		n.s.	n.s.	n.s.
	HC	0 (0; 0)	0 (0; 0)	0 (0; 0)
		n.s.	n.s.	n.s.

*NRS, numeric rating scale; EP, endometriosis patients; HC, healthy controls; IQR, interquartile range; n.s., not significant;*

a *uncorrected p-values for within-group comparison with previous phase*.

* *p ≤ 0.05;*

** *p ≤ 0.01;*

*** *p ≤ 0.001; Bonferroni-corrected p-values for comparison between groups*.

As shown in Table 3, EP reported PMDD-associated symptoms mainly during the menstruation phase, while HC did not show any PMDD symptoms during the menstrual cycle. Group comparisons revealed that EP were significantly more likely than HC to report depressed mood, anxiety, affective lability, anger/irritability, difficulty concentrating, lethargy, hypersomnia/insomnia, perceived loss of control, and physical symptoms during the menstruation phase. In addition, physical symptoms were elevated during the entire cycle in EP compared with HC (Table 3).

**Table 3:** Diary results for PMDD symptoms in endometriosis patients and healthy controls, according to cycle phases.

**PMDD symptoms (range 0–5)**	**Group (EP *n =* 12; HC *n =* 11)**	**Phase**		
		**Menstruation phase**	**Intermediate phase**	**Premenstruation phase**
Depressed mood. *median (IQR)*	EP	1.72 (1.17; 2.4)^**^	0.42 (0.11; 1.55)	1.03 (0; 1.42)
		≤ 0.05*^a^*	≤ 0.05*^a^*	n.s.
	HC	0.17 (0.11; 0.39)	0.33 (0.03; 0.7)	0.2 (0; 0.87)
		n.s.	n.s.	n.s.
Anxiety. *median (IQR)*	EP	1.83 (0.75; 2.43)^**^	0.63 (0.32; 1.06)	1.9 (1.9; 7.45)
		n.s.	≤ 0.01*^a^*	n.s.
	HC	0.2 (0; 0.77)	0.3 (0; 0.8)	0.27 (0.07; 1.2)
		n.s.	n.s.	n.s.
Affective lability. *median (IQR)*	EP	1.41 (1.11; 1.75)^**^	0.28 (0.06; 1.18)	0.6 (0; 4.4)
		≤ 0.05*^a^*	≤ 0.01*^a^*	n.s.
	HC	0.1 (0; 0.5)	0.4 (0; 0.9)	0.07 (0; 0.33)
		n.s.	n.s.	n.s.
Anger/irritability. *median (IQR)*	EP	1.95 (1.04; 3.04)	0.53 (0.16; 1.03)	0.3 (0; 1.65)
		≤ 0.01^a^^**^	≤ 0.01^a^	n.s.
	HC	0.3 (0.1; 0.65)	0.4 (0.15; 0.85)	0.5 (0.1; 1.25)
		n.s.	n.s.	n.s.
Loss of interest. *median (IQR)*	EP	2.2 (0.4; 2.58)	0.29 (0.01; 0.87)	0 (0; 0.5)
		≤ 0.05^a^	≤ 0.01^a^	n.s.
	HC	0.06 (0; 0.5)	0.32 (0.05; 0.67)	0.4 (0; 1.4)
		n.s.	n.s.	n.s.
Difficulty in concentrating *median (IQR)*	EP	2.2 (0.98; 2.8)^***^	0.65 (0.1; 1.38)	0.2 (0; 1.2)
		≤ 0.01^a^	≤ 0.01^a^	n.s.
	HC	0 (0; 1.2)	0.25 (0; 0.71)	0.2 (0; 1.2)
		n.s.	n.s.	n.s.
Lethargy *median (IQR)*	EP	2.5 (1.68; 3.19)	1.05 (0.4; 1.88)	0.95 (0; 1.9)
		≤ 0.01^a^	≤ 0.05^a^	n.s.
	HC	0.9 (0.4; 1.15)	0.5 (0.14; 1.12)	1.2 (0.5; 1.8)
		n.s.	n.s.	n.s.
Change in appetite *median (IQR)*	EP	0.93 (0.42; 1.55)	0.39 (0.12; 0.74)	0.07 (0; 0.37)
		≤ 0.05^a^	n.s.	n.s.
	HC	0.33 (0.17; 0.67)	0.26 (0.06; 0.72)	0.27 (0; 0.53)
		n.s.	n.s.	n.s.
Hypersomnia/ insomnia *median (IQR)*	EP	1.15 (0.69; 1.76)^**^	0.54 (0.14; 1.05)	0.15 (0; 0.85)
		≤ 0.01^a^	≤ 0.05^a^	n.s.
	HC	0.4 (0.3; 0.7)	0.32 (0.23; 0.71)	0.5 (0.2; 1.4)
		n.s.	n.s.	n.s.
Perceived loss of control *median (IQR)*	EP	1.85 (0.65; 2.35)^**^	0.53 (0.07; 0.97)	0 (0; 1.9)
		n.s.	≤ 0.01^a^	n.s.
	HC	0 (0; 0.6)	0.29 (0.09; 0.58)	0 (0; 0.8)
		n.s.	n.s.	n.s.
Physical symptoms *median (IQR)*	EP	1.03 (0.69; 2.02)^**^	0.55 (0.2; 1.09)^**^	1.18 (0.35; 2.22)^**^
		n.s.	≤ 0.01^a^	≤ 0.05^a^
	HC	0.23 (0.1; 0.8)	0.1 (0.06; 0.24)	0.21 (0.15; 0.4)
		n.s.	n.s.	n.s.

*PMDD, Premenstrual dysphoric disorder; IQR, interquartile range; EP, endometriosis patients; HC, healthy controls; n.s., not significant*.

a *uncorrected p-values for within-group comparison with previous phase*.

** *p ≤ 0.01;*

*** *p ≤ 0.001;*

*Bonferroni-corrected p-values for comparison between groups*.

### Qualitative Interviews

The qualitative interviews revealed that living with endometriosis is associated with impairment in everyday life. Some women described not being completely free of symptoms on any given day and that their symptoms increased around menstruation. Many interests or other needs were subordinated to the disease. The following three main categories were identified: impact on a physical level, a mental level, and a social level (see Table 4): In addition to severe pain, women reported many other physical symptoms, e.g., gastrointestinal and circulatory problems, heavy bleeding, migraine, tightness in the chest, up to a strong physical exhaustion. Most women described severe mental distress, especially the perceived loss of control due to various physical symptoms that often occurred unpredictably, and the limited treatment options for endometriosis. Moreover, women described a constant mental preoccupation with pain. Living with endometriosis affected their personality and they no longer felt like themselves. Patients reported impaired social interactions (e.g., their intimate relationships), restricted participation in daily life (e.g., travel or participating at events) and impaired occupational performance. As a result, avoidance behavior was reported, and patients increasingly withdrew in order to recover.

**Table 4:** Interview analysis guide and examples.

**Category**	**Definition**	**Anchor examples**
Impact on physical level	•All physical symptoms perceived by the women: ° Pain, migraine, strong menstrual bleeding, etc. ° Sleeping problems ° Impaired functionality and activity	•“*So for me, it was primarily the pain”* •“*...always strong cramps, also at the sphincter and also strong diarrhea and nausea”* •“*...I just also have a bleed, that I know a sanitary towel and a tampon, they're both not going to hold anymore”*
Impact on mental level (Impact on personality)	•Impact on emotions and thoughts •Restrictions in concentration, load capacity, and “scope of action” •Awareness of limited treatment options •Occurrence of different, changing physical symptoms	•“*…most of all the psychological strain, actually, that I can't plan”* •“*so I am really there mega dissatisfied and also really desperate. And I've never had feeling I've never had before...”* •“*and the not really existing conventional medical options, because you just don't have that much perspective in a certain way”*
Impact on social level (professional level)	•Avoidance of social events, traveling etc. (staying at home) •Impact on social relationships (intimate partnership) •Impact on practicing the women's professions	•“*...my friend, that excites him, he can understand it a little bit, but for him it's also bad”* •“*And what also bothers me a lot is, that I isolate myself so much from other people”* •“*And also at work, that bothers that I'm just not as resilient as I used to be”* •“*And I couldn't go home by myself anymore either, my colleagues had to drive me home”*

*Interviews have been conducted in German*.

## Discussion

Endometriosis is a chronic disease that is often associated with pelvic pain. However, this does not do justice to the day-to-day reality of women with endometriosis, who are confronted with variety of symptoms that often appear suddenly and interact with each other. The aim of the present study was to gain a holistic understanding of the everyday reality of women with endometriosis. For this purpose, a mixed-methods design was used to collect information on different levels and relate them to each other.

The standardized questionnaires yielded worse results for the patients compared to healthy controls. Accordingly, endometriosis patients showed significantly higher depression and anxiety scores (HADS) during menstruation, as well as a reduced mental and physical quality of life (SF-12, FW-7), more stress as well as more physical symptoms (PHQ-15). This is not only in accordance with previous studies (18, 20, 48, 49), but was further confirmed by the detailed diary survey, which provided the opportunity to observe symptom fluctuations over the entire menstrual cycle. By pseudonymizing the diary entries, biases induced by social desirability could be avoided. It was very impressively demonstrated that patients reported significantly more pelvic pain (up to 80 on NRS 0-100) and further physical symptoms throughout the menstrual cycle. During menstruation all PMDD associated symptoms (except for changes in appetite) such as depression, anxiety, loss of control, or loss of concentration, were significantly increased, indicating that endometriosis patients have to battle with many other physical and psychological stresses in addition to pain. PMDD symptoms were not elevated during the premenstrual phase in either EP or HC, suggesting that PMDD *per se* has not occurred in this cohort, which is in agreement with the exclusion criterion of mental illness. Nevertheless, patients showed the highest levels of physical symptoms during the premenstrual period.

In order to avoid the sole evaluation of a priori predefined categories and to offer the opportunity to speak freely and openly about their experiences, semi-structured interviews were conducted with the endometriosis patients. The face-to-face interviews showed that living with endometriosis was associated with manifold impairment in everyday life. Physical strains, especially pain, high levels of psychological distress, and social limitations were reported. Living with endometriosis affected the women's personality and they “no longer felt like themselves.” The constant physical, emotional, and cognitive preoccupation with pain appears to result in a number of other mental and social negative consequences. The recognition that, in addition to other physical symptoms, pain serves as a kind of “starting point” for a whole series of associated and mutually influencing mental and social strains has also been described in previous studies (5, 20, 28, 49, 50). Furthermore, discomfort fluctuated over time, sometimes unpredictably, creating tremendous uncertainty and avoidance behavior. In their systematic review, Gao et al. identified pain, psychosocial functioning, and social functioning as particularly affected domains in women with endometriosis, and the present results further support this conclusion. However, in the present study, the influence of other fluctuating physical symptoms (e.g., nausea or exhaustion) across the entire cycle became additionally evident (51).

The current study is limited by the relatively small and heterogenous sample; a larger sample size would have been desirable. Especially time since diagnosis varied considerably among patients [on average 41.92 months (±17.74)]. In addition, disease-unrelated factors (e.g., disputes or professional challenges) might have influenced the participants' daily condition and for instance might have led to concentration difficulties or anxiety. The complex and comprehensive data ensured a holistic picture of participants' experiences, but certainly also led to fatigue and habituation effects due to the extensive questionnaires. Although the completion of the diary was sometimes perceived as exhausting by the participants, it was nevertheless manageable for them as it was predictable and time-limited.

In conclusion, the multi-method approach of this study revealed that pain and the associated cognitive and emotional distress, as well as the unpredictable occurrence of symptoms, led to significant impairment at the physical, mental, and social levels in women with endometriosis; this process appears to be reinforced by the lack of a curative therapy. Similar to other chronic pain conditions (52), therapeutic approaches that perceive the disease from a bio-psycho-social perspective should be applied to reduce the negative impact of endometriosis on women's daily lives. Accordingly, a multimodal treatment approach is warranted that includes not only drug treatment and surgery, but also addresses the bio-psycho-social needs of the patients (53). Future studies with larger samples and longer observation times are needed to better understand the fluctuations of daily symptoms in endometriosis patients over time as well as the influence of individual factors, such as type of endometriosis, treatment approach, and psychosocial stressors.

## Data Availability Statement

The raw data supporting the conclusions of this article will be made available by the authors, without undue reservation.

## Ethics Statement

The studies involving human participants were reviewed and approved by Ethical Committee of the Medical Faculty at LMU Munich (no. 17-695). The patients/participants provided their written informed consent to participate in this study.

## Author Contributions

EO, AW, VH, RMP, and KM contributed to conception and design of the study. EO and KM performed the statistical analysis and wrote the first draft of the manuscript. All authors contributed to manuscript revision, read, and approved the submitted version.

## Funding

KM received a grant from the Schweizer-Arau-Foundation, Germany.

## Conflict of Interest

The authors declare that the research was conducted in the absence of any commercial or financial relationships that could be construed as a potential conflict of interest.

## Publisher's Note

All claims expressed in this article are solely those of the authors and do not necessarily represent those of their affiliated organizations, or those of the publisher, the editors and the reviewers. Any product that may be evaluated in this article, or claim that may be made by its manufacturer, is not guaranteed or endorsed by the publisher.
